# Clinical assessment versus radiological investigations: Predictive accuracy for intra-abdominal injury after blunt trauma

**DOI:** 10.6026/973206300222675

**Published:** 2026-04-30

**Authors:** Dheeraj Kain, Vishal Patil, Nitin Patta, Rishi Kumar Athya, Richali Gujare, Akansha Sahare, Sachin Parmar

**Affiliations:** 1 Department of Gastrointestinal Surgery Bhopal Memorial Hospital and Research centre Bhopal, Madhya Pradesh, India; 2 Department of Surgical Oncology Bhopal Memorial Hospital and Research centre Bhopal, Madhya Pradesh, India; 3 Department of General Surgery, Shri Aurobindo Institute of medical sciences, Indore, Madhya Pradesh, India; 4 Department of Obstetrics and Gynaecology, Mahaveer Medical College, Bhopal, Madhya Pradesh, India; 5 Department V.K.S. Government Medical College, Neemuch, Madhya Pradesh, India

**Keywords:** Blunt abdominal trauma, clinical assessment, FAST ultrasound

## Abstract

Blunt abdominal trauma remains a critical biomedical problem because clinical examination alone has limited sensitivity and negative predictive value for detecting intra abdominal injuries in hemodynamically stable patients. Therefore, it is of interest to measure the clinical assessment of the diagnostic value of radiological versus clinical investigation with 100 patients with blunt abdominal trauma aged between 20-60 years in a tertiary care center in Rewa, India (April 2021- June 2022). Most prevalent was road traffic accidents (50%), followed by spleen (25%), clinical signs were pain (98%) and tenderness (90%), with the USG/X-ray (100%) and CECT (60%) leading to 70% conservative treatment with 94% good and 6% death. Thus, the radiological imaging proved to be a better diagnostic tool than clinical assessment of intra-abdominal injuries.

## Background:

Clinical evaluation cannot detect intra-abdominal injuries in stable haemodynamic patients, especially the injured [1]. In polytrauma, distracting injuries, obesity and altered mental status can mask abdominal tenderness, guarding or distension, which have low visceral damage detection sensitivity (about 50) [2]. According to Achatz *et al.* [3], clinical assessment was used for initial evaluation, but its low negative predictive value requires additional imaging to prevent missed injuries, which cause most traumatic deaths. In a similar study, Deunk *et al.* [4] found that selective CT after clinical and plain radiographic examination reveals unexpected pathologies in over 70% of blunt trauma patients and changes management in a third. Due to its high sensitivity (95-100%) and specificity (98-100%) of solid organ and hollow viscus trauma, multidetector computed tomography (MDCT) is the new standard for blunt abdominal trauma assessment [5]. Drasin *et al.* [6] state that MDCT can accurately describe injury grade, detect active extravasation and detect free fluid without solid organ injury, but 3% of blunt trauma patients with isolated intraperitoneal fluid should be examined further. Çaylak *et al.* [7] confirm prediction rules like PECARN, which uses clinical variables and imaging to avoid unnecessary CT scans and has a 97% sensitivity rate for intra-abdominal injury. Bode *et al.* [8] show how sonography in early algorithms can detect free fluid with 88% sensitivity and 100% specificity, enabling rapid patient triage with 99% accuracy on 1,671 patients. Bedside use of focused assessment with sonography for trauma (FAST) is limited to hemoperitoneum detection, with sensitivities of 77-88% and specificities over 98% [9, 10]. FAST is a useful screening tool despite its high operator dependence, according to Kumar *et al.* meta-analysis [11] 82% sensitivity and 99% specificity for adult intra-abdominal injury detection. In screening blunt trauma patients, Nural *et al.* [12] note ultrasonography's overall diagnostic performance (sensitivity 86% specificity 97) and recommend using it in conjunction with clinical judgement to make the initial diagnosis. Miele *et al.* [13] add contrast-enhanced ultrasound (CEUS), which has a positive predictive value of 98.8% and a negative predictive value of 94.1, comparable to CT in stable patients and without radiation. Diagnostic differences emphasise the need for a multimodal approach, where clinical evaluation triages unstable patients for laparotomy and imaging manages stable patients [14]. Nnamonu *et al.* [10] confirm that abdominal US is useful in resource-constrained areas and that free fluid scanning is better than organ-specific views. Fabig *et al.* [9] state that FAST cannot detect clinically significant injury early, supporting CT's dominance despite radiation. According to Lentz *et al.* [15], ultrasound can detect intraperitoneal fluid with 87% sensitivity and 96% accuracy, making it a frontline tool. It changes clinical examination from a primary diagnostic method to a triage adjunct in an imaging-centered, multimodal strategy for stable patients in similar tertiary care settings. Therefore, it is of interest to describe the Diagnostic value of clinical assessment vs radiological Investigations in blunt trauma abdomen.

## Materials and Methods:

This was a prospective observational study entitled Diagnostic value of clinical assessment versus radiological investigations in abdomen blunt traumas, which was done in Department of Surgery at Shyam Shah Medical College and Associated Sanjay Gandhi Memorial Hospital, Rewa (Madhya Pradesh). It was approved by the institutional ethics committee. The research period was between 1st April 2021 and 30th June 2022, which is duration of 15 months.

## Study design:

All patients admitted to the emergency and routine units of the General Surgery with blunt abdominal trauma and aged between 20 and 60 years were included in the study. This was aimed to compare clinical and radiological parameters that influence the decisions of emergency management. Patients who provided informed written consent were recruited only.

## Inclusion criteria:

[1] Blunt abdominal trauma patients who are between 20 and 60 years old.

[2] Patients with and without comorbidities.

[3] Patients and their attendants who will sign the written informed consent.

## Exclusion criteria:

[1] Patients and attendants that is not willing to give written consent.

[2] Patients under 20 years and more than 60 years.

[3] Frail- Trauma patients other than blunt abdominal trauma.

## Patient data collection:

Upon admission, all the patients received instant clinical examination, such as checking the color of skin, the degree of consciousness, pulse quality, blood pressure, respiratory rate and respiratory pattern, body temperature states and pupil state. The first aid treatment was immediately initiated by getting intravenous access and taking blood samples to check complete blood count, blood grouping and cross- matching, blood sugar, urea and other biochemical parameters. Microscopic hematuria, sugar and albumin examination in the urine were done. Those patients that failed to respond to fluid resuscitation were placed in Trendelenburg position and subjected to steroids and vasopressors (e.g., dopamine infusion) until stable. Abdominal paracentesis using four quadrants was done randomly in patients to facilitate diagnosis. The diagnostic peritoneal lavage was used in cases of negative or inconclusive paracentesis. Conventional imaging comprised erect chest and abdominal X-rays, to diagnose rib fractures, pneumothorax, hemothorax, gas under diaphragm, splenic shadow abnormalities, gastric indentation, haziness, psoas shadow obliteration and lower rib fractures.

## Clinical examination:

A systematic case pro forma was used to record detained history taking and comprehensive general and local abdominal examination of each patient. The abdominal signs (tenderness, guarding, distension, rigidity and bowel sounds) received special attention.

## Laboratory investigations:

[1] Hemoglobin estimation

[2] Total and differential counts of leukocytes.

[3] Blood urea and serum creatinine levels.

[4] Liver function tests

[5] Cross-matching and blood grouping.

[6] Microscopic hematuria, sugar and albumin examination of the urine.

## Radiological examination:

[1] Chest X-ray: Assessed to check the presence of rib fractures, pneumothorax and hemothorax.

[2] Abdominal X-ray (Erect posture): Evaluated based on presence of gas under diaphragm, splenic shadow variances, gastric induration, generalized haziness, psoas shadow obliteration, lower rib fracture.

## Abdominal ultrasound:

Examine to identify the presence or enlargement of the splenic hematoma, free intraperitoneal fluid, kidney and bladder injuries and retroperitoneal hematoma. All clinical and radiological results were compared with each other to diagnose and make decisions regarding management.

## Results and Discussion:

One hundred patients with blunt abdominal trauma were studied and their mean age was found to be 38.44/17.396 years with majority (26) falling between age group 21-30 years (Table 1 - see PDF). Road traffic accidents (50%), falls from height (20%) and blows with blunt objects (30) were the major etiologies whereas common clinical features were pain (98%), tenderness (90%), vomiting (50%), abdominal distension (38%), guarding (40%), absent bowel sounds (36%), hemoglobin less than 10 g/dL (22%) and shock (18%) were the common etiologies and common clinical features respectively (Table 2 and Table 3 - see PDF) The most common affected organ was the spleen (25%), liver and small intestine (10% each), large intestine/rectum and urinary bladder (6 and 4% each), kidney (4%), pancreas (2%) and mesentery/ retroperitoneum (1% each) (Table 4 - see PDF) and diagnostic modalities were X-ray and USG (100 and 100% respectively) and CECT (60% each) (Table 5 - see PDF). The conservative (70) and operative (30) management, with operative procedures of resection and anastomosis (16.66%), splenectomy (10%) and bladder repair (6.66%) had better management with the better outcome of 94 percent and mortality at 6 percent and 30 percent respectively and hospital stays of 8 days and less (50%), 8-14 days (30%), 15-21 days (18%) and over 21 days (Table 6 - see PDF).

In the present study, blunt abdominal trauma predominantly affected young to middle-aged adults with male preponderance (56%), which aligns with Mirzamohamadi *et al.* [16] who reported similar demographics in a large Iranian trauma cohort where males constituted over 70% of abdominal injury cases. Qureshi *et al.* [17] also reported similar demographics 69 male (67%) and 34 female (33%) whose ages were between 18 and 80 years (mean age 42.6 ± 16.3 years).Road traffic accidents as the leading cause (50%) mirror findings by Yadollahi *et al.* [18] in a level I trauma center, where vehicular trauma accounted for 45-55% of blunt abdominal injuries, reflecting high-velocity mechanisms prevalent in developing regions. Clinical features like pain (98%) and tenderness (90%) were universal, consistent with O'Rourke *et al.* who emphasize these as primary screening signs despite limited specificity in blunt trauma evaluation [1]. Spleen emerged as the most common organ injured (25%), followed by liver (10%), Larsen *et al.* [19] who identified splenic trauma in 30-40% of blunt solid-organ cases due to its vascular fragility and left-upper quadrant vulnerability. This distribution also matches Dalton *et al.* [20] with spleen and liver comprising over 50% of injuries in their series, though minor variations may stem from referral patterns as noted by Arumugam *et al.* [21] Bowel and bladder injuries (16% combined) align with Ghimire *et al.* [22] from Nepal, reporting hollow viscus involvement in 10-20% of blunt abdominal trauma admissions. Universal use of USG and X-ray with selective CECT (60%) reflects standard triage, as Federle *et al.* [23] advocate CT as the gold standard for stable patients to grade injuries and guide therapy, with FAST/USG enabling rapid initial assessment. The 70% non-operative management (NOM) success rate echoes Cimbanassi *et al.* [24] from the International Consensus Conference, who reported 85-95% NOM feasibility for stable splenic and hepatic injuries, reducing operative risks. Ibrahim *et al.* [25] similarly achieved high NOM rates (over 80%) in their prospective study, attributing success to hemodynamic monitoring, while Velmahos *et al.* [26] confirmed durable outcomes in 90% of conservatively managed solid-organ trauma. Postoperative complications like surgical site infection (18%) and respiratory issues (8%) are comparable to Ibrahim *et al.* [25] who observed similar morbidity profiles in surgically managed cases from Egypt, underscoring NOM's role in minimizing laparotomy-related burdens. The low mortality (6%) and short hospital stays (50% <8 days) parallel Kanlerd *et al.* [27], linking favorable outcomes to protocolized NOM and timely intervention. Overall, these findings reinforce Mirzamohamadi *et al.* [16] and Yadollahi *et al.* [18] on optimized imaging-driven management yielding low mortality in resource-limited settings.

## Conclusion:

Radiological investigations, particularly USG and CECT, provide superior diagnostic accuracy compared to clinical assessment alone in detecting intra-abdominal injuries following blunt abdominal trauma, enabling precise triage and management decisions. Spleen was the most commonly injured organ (25%), with road traffic accidents as the predominant etiology (50%), supporting non-operative management in 70% of hemodynamically stable patients achieving 94% favorable outcomes. Early imaging integration with clinical evaluation optimizes resource utilization and minimizes missed injuries in resource-limited settings. Routine protocolized screening remains essential to reduce mortality (6%) and complications in blunt abdominal trauma.
